# Trends and socio‐economic disparities in chronic lymphocytic leukaemia 2014–2022: An England‐wide cohort study from the UNCOVER group

**DOI:** 10.1111/bjh.70765

**Published:** 2026-08-17

**Authors:** Temitope Erinfolami, Amelia Fisher, Indrani Karpha, Yeong Lim, Nurunnahar Akter, Toyyib Abdulkareem, Brogan Johnston, Catrin Tudur Smith, Hilary Lindsay, Shaun Bowden, Debbie Yates, Lelia Duley, Renata Walewska, Stella Williams, David Allsup, Nagesh Kalakonda, Mark Bishton, Andrew Pettitt, Nicolas Martinez‐Calle

**Affiliations:** ^1^ University of Liverpool Liverpool UK; ^2^ University of Leeds Leeds UK; ^3^ Leeds Teaching Hospitals NHS Trust Leeds UK; ^4^ Clatterbridge Cancer Centre NHS Foundation Trust Liverpool UK; ^5^ CLL Support Association Chippenham UK; ^6^ University Hospitals of Dorset NHS Foundation Trust Bornemouth UK; ^7^ Centre for Biomedicine, Hull York Medical School University of Hull Hull UK; ^8^ Nottingham University Hospitals NHS Trust Nottingham UK; ^9^ University of Nottingham Nottingham UK

**Keywords:** cause‐specific survival, chronic lymphocytic leukaemia, CLL, deprivation, epidemiology, incidence, overall survival, population‐based

## Abstract

To re‐evaluate chronic lymphocytic leukaemia (CLL) epidemiology in the era of modern therapy, data were extracted from the English National Cancer Registration Dataset (NCRD). A total of 34 427 consecutive diagnoses of CLL were recorded (2014–2022), the largest European dataset on record. In addition to known associations with age, gender‐ethnicity, multivariable analysis showed incidence rate ratio (IRR) variations with government region (IRR ranging from 0.78 to 0.94 vs. London), socioeconomic deprivation (1.06 [95% confidence interval (CI) 1.02–1.10] for most vs. least deprived quintile) and IRR fall in successive calendar years (0.70 [95% CI 0.67–0.73] for 2022 vs. 2014). With a median follow‐up of 74.4 months, 36.9% of patients had died, including 13.6% from blood cancer. 1‐, 3‐ and 5‐year overall survival (OS)/net survival rates were 90%/94%, 78%/87% and 67%/81%, respectively. Age, male gender, comorbidity and socioeconomic deprivation were independently associated with shorter OS and cause‐specific survival (CSS), with OS‐hazard ratio (HR) of 1.42 (95% CI 1.34–1.50) and CSS‐HR of 1.28 (1.16–1.41) for the most versus least deprived quintiles. In addition, government region was independently associated with CSS, with HRs relative to London ranging from 0.84 (0.74–0.96) to 1.12 (0.99–1.27). Our findings reveal previously unreported disparities in CLL incidence and survival based on place of residence.

## INTRODUCTION

Chronic lymphocytic leukaemia (CLL) is a chronic B‐cell malignancy considered incurable and requiring multiple lines of treatment during the course of the disease.^1^, ^2^ Life expectancy can be close to 30 years in patients diagnosed in their 5th decade. Up to 80% of patients in European populations^3^ are diagnosed incidentally with early‐stage disease and can remain on active monitoring for a prolonged time. Untreated CLL patients are known to experience progressive immunosuppression and are at higher risk of infections, including coronavirus disease 2019 (COVID‐19), compared to age‐ and sex‐matched populations.^4^

Previously available data reported an average of 3952 new CLL cases per year in the United Kingdom between 2017 and 2019,^5^ making it the most commonly diagnosed haematological malignancy. Despite the step‐change in CLL therapy that has taken place over the last decade with the replacement of chemoimmunotherapy by small‐molecule inhibitors with or without anti‐CD20 monoclonal antibodies (anti‐CD20 mAbs), there is a paucity of UK studies describing CLL epidemiology in the era of novel agents.

Understanding the impact of COVID‐19 in people with blood cancer (UNCOVER) is a blood cancer health data research programme that utilises linked data in the NHS England National Cancer Registration Dataset (NCRD). The latter includes a wide range of data on all patients diagnosed with all types of cancer in all NHS institutions in England.^6^ Consequently, UNCOVER has the potential to add significantly to existing information and knowledge on CLL generated from population‐based registries in other countries.^7^, ^8^, ^9^, ^10^, ^11^, ^12^

Here we present the first report from the UNCOVER CLL Work Package. We establish the true incidence of CLL in England between 2014 and 2022, describe the population's baseline characteristics, report its overall and net survival, and identify relevant associations with both incidence and survival.

## METHODS

### Data sources

Anonymised NCRD data for all patients in England diagnosed with any type of blood cancer between January 2014 and December 2022 were extracted and transferred to the University of Liverpool for analysis in the UNCOVER health data research programme (Research Ethics Committee approval #21/WA/0114). NHS organisations have a statutory requirement to record all new cancer diagnoses into NCRD using International Classification of Diseases for Oncology, 3rd Edition (ICD‐O‐3) codes, along with a range of demographic information, date of diagnosis, Charlson comorbidity index (CCI) and indices of multiple deprivation (IMD). The NCRD is also linked to mortality data from the Office for National Statistics (ONS), which provides the date of death or last follow‐up and the cause of death on the death certificate recorded using International Classification of Diseases, 10th Revision (ICD‐10) codes. Follow‐up data were available from the date of first diagnosis until death recorded in the cancer registry, loss to follow‐up or 5 July 2023, whichever was the earliest.

### Study population

All patients diagnosed with CLL aged 18 years and older were identified using the ICD‐O‐3 diagnostic codes 98233 and 96703 (Table S1). Patients with a CLL diagnosis recorded on death certification only (DCO) were excluded.

### Variables

Demographic variables analysed include age at diagnosis (in years and categorised into age groups); gender; ethnicity; government region (Figure S1); calendar year of diagnosis; CCI based on a lookback from 78 to 6 months before diagnosis^13^; and IMD quintile. IMD is the official measure of relative deprivation for small local areas in England and is based on income, education, employment, health, crime, accessibility of housing and local services, and living environment.^14^ IMD was calculated using the 2019 published indices at the Lower Super Output Area (LSOA) level and categorised into national quintiles (Q1 = living in the most deprived LSOAs; Q5 = living in the least deprived LSOAs).^15^ For age‐adjusted incidence analysis, population‐weighted interpolation was used to harmonise 2021–2022 population estimates to LSOA boundaries and align with the geography of the IMD.

### Statistical analyses

Age‐standardised incidence rates (ASIRs) were calculated as the number of new cases per 100 000 English population, using the European Standard Population (2013) to account for the age structures in the population over time and between groups.^16^ A Poisson regression model was used to estimate correlations of incidence rate ratios (IRRs) with selected patient characteristics (except comorbidity). All incidence analyses used the estimates of the mid‐year population of England as the denominator. These estimates were obtained from the ONS.^17^

Mortality records were obtained from the medical certificate of cause of death (MCCD). In the United Kingdom, part 1 of the death certificate lists the direct causes of death, whereas part 2 lists co‐morbidities and contributors that influence the sequence of events in part 1.^18^ The National Disease Registration Service (NDRS) provides an underlying cause of death, which is a deterministic assignment based on information reported in the MCCD.

Overall survival (OS) was estimated using the Kaplan–Meier method, and multivariable Cox proportional hazards models were fitted to examine the association between hazard of death and demographic/clinical variables (age, gender, IMD quintile, region, ethnicity, year of diagnosis and CCI). Patients alive at the last follow‐up date were censored. Net survival (NS) was calculated using the Pohar‐Perme estimator^19^, ^20^ using expected mortality rates^21^ stratified by age, gender, IMD quintile and government region obtained from subnational life tables.^22^

Cause‐specific survival (CSS) was used to estimate the probability of blood cancer death in the presence of competing risks. A multivariable cause‐specific Cox model was fitted to examine the associations between CSS and the same covariates listed above. To account for errors or imprecision in nomenclature on the death certificate and capture cases of Richter transformation and secondary myelodysplastic syndrome (MDS) or acute myeloid leukaemia (AML), all deaths attributed to any form of blood cancer were considered to be CLL‐related. Deaths from other causes were censored.

Multivariable analyses were restricted to cases with complete information; no imputation for missing data was performed. The proportional hazards assumption for the Cox models was checked using Schoenfeld residuals. All analyses were conducted using R software, version 4.4.1,^23^ with a two‐sided 5% significance threshold used for all statistical tests.

## RESULTS

### Patient characteristics

A total of 34 427 patients meeting the study criteria were identified (Figure 1). As expected, the cohort comprised nearly twice as many males as females and included only a small percentage of people of non‐White ethnicity. Median age was 72 years (range: 18–107; interquartile range: 64–80) (Table 1). In keeping with this, more deprived IMD quintiles, which are associated with shorter life expectancy,^24^ were under‐represented, whereas less deprived quintiles were over‐represented (Table 1). Information on route‐to‐diagnosis (collected until 2018) was available for 16 815 (48.8%) patients, of whom 52.0% were diagnosed following routine general practitioner (GP) referrals, 24% via urgent ‘Two‐Week Wait’ (TWW) GP referrals and 14.3% following an Emergency Presentation (Table 1, Figure S3). There was no significant regional variation observed in the route to diagnosis (Table S2).

**FIGURE 1:**
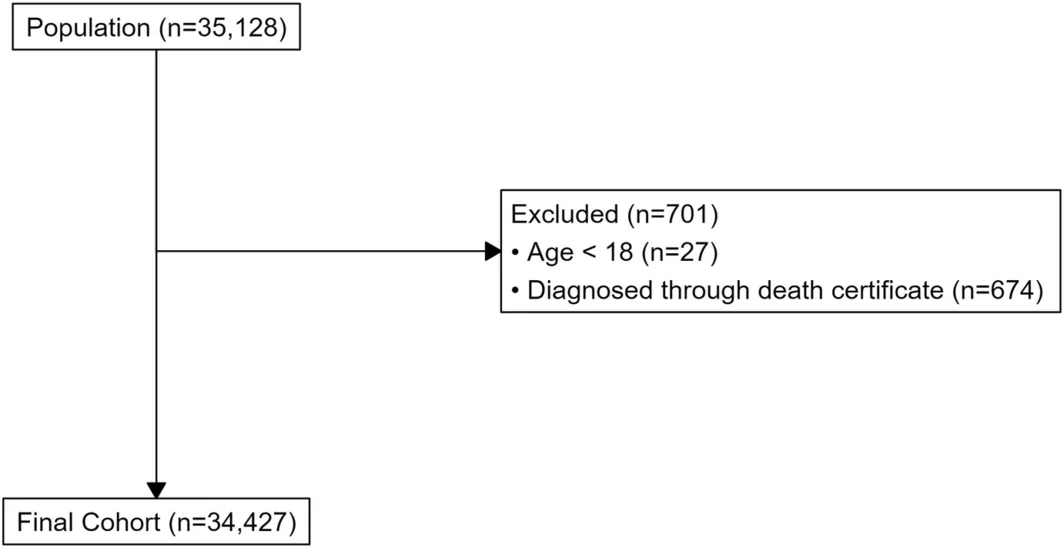
PRISMA diagram showing patient selection.

**TABLE 1:** Characteristics of patients diagnosed with CLL in England, 2014–2022.

Variable	2014–16	2017–19	2020–22	Overall
	*N* = 12 100	*N* = 11 709	*N* = 10 618	*N* = 34 427
Gender				
Female	4553 (38%)	4384 (37%)	3938 (37%)	12 875 (37%)
Male	7547 (62%)	7325 (63%)	6680 (63%)	21 552 (63%)
Age (grouped)				
18–44	171 (1.4%)	195 (1.7%)	187 (1.8%)	553 (1.6%)
45–54	805 (6.7%)	790 (6.7%)	691 (6.5%)	2286 (6.6%)
55–64	2174 (18%)	2074 (18%)	1930 (18%)	6178 (18%)
65–74	3883 (32%)	3858 (33%)	3361 (32%)	11 102 (32%)
75+	5067 (42%)	4792 (41%)	4449 (42%)	14 308 (42%)
Ethnicity				
White	10 755 (89%)	10 312 (88%)	8695 (82%)	29 762 (86%)
Mixed	39 (0.3%)	41 (0.4%)	38 (0.4%)	118 (0.3%)
Asian	240 (2.0%)	263 (2.2%)	222 (2.1%)	725 (2.1%)
Black/Caribbean	128 (1.1%)	133 (1.1%)	123 (1.2%)	384 (1.1%)
Other	123 (1.0%)	148 (1.3%)	115 (1.1%)	386 (1.1%)
Not known	815 (6.7%)	812 (6.9%)	1425 (13%)	3052 (8.9%)
Government region				
London	1266 (10%)	1143 (9.8%)	1029 (9.7%)	3438 (10.0%)
North East	675 (5.6%)	653 (5.6%)	674 (6.3%)	2002 (5.8%)
North West	1692 (14%)	1660 (14%)	1578 (15%)	4930 (14%)
Yorkshire and The Humber	1271 (11%)	1318 (11%)	1156 (11%)	3745 (11%)
East Midlands	1172 (9.7%)	1106 (9.4%)	1068 (10%)	3346 (9.7%)
West Midlands	1246 (10%)	1282 (11%)	1075 (10%)	3603 (10%)
East of England	1392 (12%)	1437 (12%)	1120 (11%)	3949 (11%)
South East	1939 (16%)	1807 (15%)	1685 (16%)	5431 (16%)
South West	1447 (12%)	1303 (11%)	1233 (12%)	3983 (12%)
IMD quintile				
5—least deprived	2776 (23%)	2719 (23%)	2445 (23%)	7940 (23%)
4	2777 (23%)	2640 (23%)	2460 (23%)	7877 (23%)
3	2517 (21%)	2524 (22%)	2254 (21%)	7295 (21%)
2	2224 (18%)	2018 (17%)	1816 (17%)	6058 (18%)
1—most deprived	1806 (15%)	1808 (15%)	1643 (15%)	5257 (15%)
Charlson comorbidity index				
0	8528 (70%)	7949 (68%)	7174 (68%)	23 651 (69%)
1	1648 (14%)	1690 (14%)	1527 (14%)	4865 (14%)
2	1049 (8.7%)	1051 (9.0%)	911 (8.6%)	3011 (8.7%)
3	428 (3.5%)	534 (4.6%)	528 (5.0%)	1490 (4.3%)
4+	447 (3.7%)	485 (4.1%)	478 (4.5%)	1410 (4.1%)
Route to diagnosis^a^				
Emergency presentation	1514 (13%)	887 (7.6%)	0 (0%)	2401 (7.0%)
General Practitioner referral	5694 (47%)	3043 (26%)	0 (0%)	8737 (25%)
Inpatient elective	90 (0.7%)	127 (1.1%)	0 (0%)	217 (0.6%)
Other outpatient	951 (7.9%)	474 (4.0%)	0 (0%)	1425 (4.1%)
Two‐week wait	2321 (19%)	1714 (15%)	0 (0%)	4035 (12%)
Unknown	1530 (13%)	5464 (47%)	10 618 (100%)	17 612 (51%)

Abbreviations: CLL, chronic lymphocytic leukaemia; IMD, indices of multiple deprivation.

^a^ Route to diagnosis was not collected after 2018.

### Incidence analysis

ASIR was 6.9/100 000 population for the overall cohort, 9.4/100 000 in males and 4.8/100 000 in females, with marked differences observed between people of White (7.0/100 000) and non‐White (0.9–2.5/100 000) ethnicity (Table 2). ASIR increased with age and varied significantly across government regions, ranging from 8.3/100 000 in the North East to 6.1/100 000 in London. ASIR also decreased slowly over time until 2020, when there was a sharp drop corresponding to the first year of the COVID‐19 pandemic (Figure S2).

**TABLE 2:** Patient distribution and age‐standardised incidence rates (ASIR) with 95% confidence intervals of CLL in England, 2014–2022.

Variable	*N* (%)	ASIR [95% CI]
All Patients	34 427 (100%)	7.3 [7.1, 7.5]
Gender		
Female	12 875 (37%)	5.1 [4.9, 5.2]
Male	21 552 (63%)	9.9 [9.6, 10.2]
Age group		
18–44	553 (1.6%)	—
45–54	2286 (6.6%)	—
55–64	6178 (18%)	—
65–74	11 102 (32%)	—
75+	14 308 (42%)	—
Ethnicity		
White	29 762 (86%)	7.4 [7.2, 7.5]
Mixed	118 (0.3%)	1.0 [0.6, 1.4]
Asian	725 (2.1%)	2.1 [1.8, 2.4]
Black/Caribbean	384 (1.1%)	2.6 [2.1, 3.2]
Other	386 (1.1%)	4.8 [3.9, 5.9]
Region	3052 (8.9%)	
London	3438 (10.0%)	6.5 [6.1, 6.9]
North East	2002 (5.8%)	8.3 [7.6, 9.1]
North West	4930 (14%)	7.8 [7.4, 8.3]
Yorkshire and The Humber	3745 (11%)	8.0 [7.5, 8.5]
East Midlands	3346 (9.7%)	7.9 [7.4, 8.4]
West Midlands	3603 (10%)	7.2 [6.7, 7.7]
East of England	3949 (11%)	7.1 [6.6, 7.5]
South East	5431 (16%)	6.7 [6.3, 7.1]
South West	3983 (12%)	7.2 [6.8, 7.7]
IMD quintile		
5—least deprived	7940 (23%)	7.3 [7.0, 7.6]
4	7877 (23%)	7.4 [7.1, 7.8]
3	7295 (21%)	7.4 [7.0, 7.7]
2	6058 (18%)	7.1 [6.7, 7.5]
1—most deprived	5257 (15%)	7.2 [6.8, 7.6]
Diagnosis year		
2014	3993 (12%)	8.1 [7.6, 8.6]
2015	4093 (12%)	8.2 [7.7, 8.7]
2016	4014 (12%)	7.9 [7.4, 8.4]
2017	3874 (11%)	7.5 [7.0, 8.0]
2018	3901 (11%)	7.4 [7.0, 7.9]
2019	3934 (11%)	7.4 [6.9, 7.8]
2020	3274 (9.5%)	6.1 [5.6, 6.5]
2021	3583 (10%)	6.6 [6.2, 7.0]
2022	3761 (11%)	6.8 [6.4, 7.3]

Abbreviations: CLL, chronic lymphocytic leukaemia; IMD, indices of multiple deprivation.

Similar findings were obtained in the Poisson regression model (Figure 2). IRRs were 1.94 (95% CI 1.89–1.98) for males versus females; 104 (95–114) for age ≥75 versus 18–44; 0.13 (0.11–0.16), 0.28 (0.26–0.30) and 0.35 (0.32–0.39) for Mixed, Asian and Black versus White ethnicity, respectively; and 0.70 (0.67–0.74) for year 2022 vs. 2014. IRRs were also lower for all provincial government regions versus London (e.g. IRR 0.78 [0.74–0.82] for the South East) and higher for the most versus least deprived IMD quintile (IRR 1.06 [1.02–1.10]).

**FIGURE 2:**
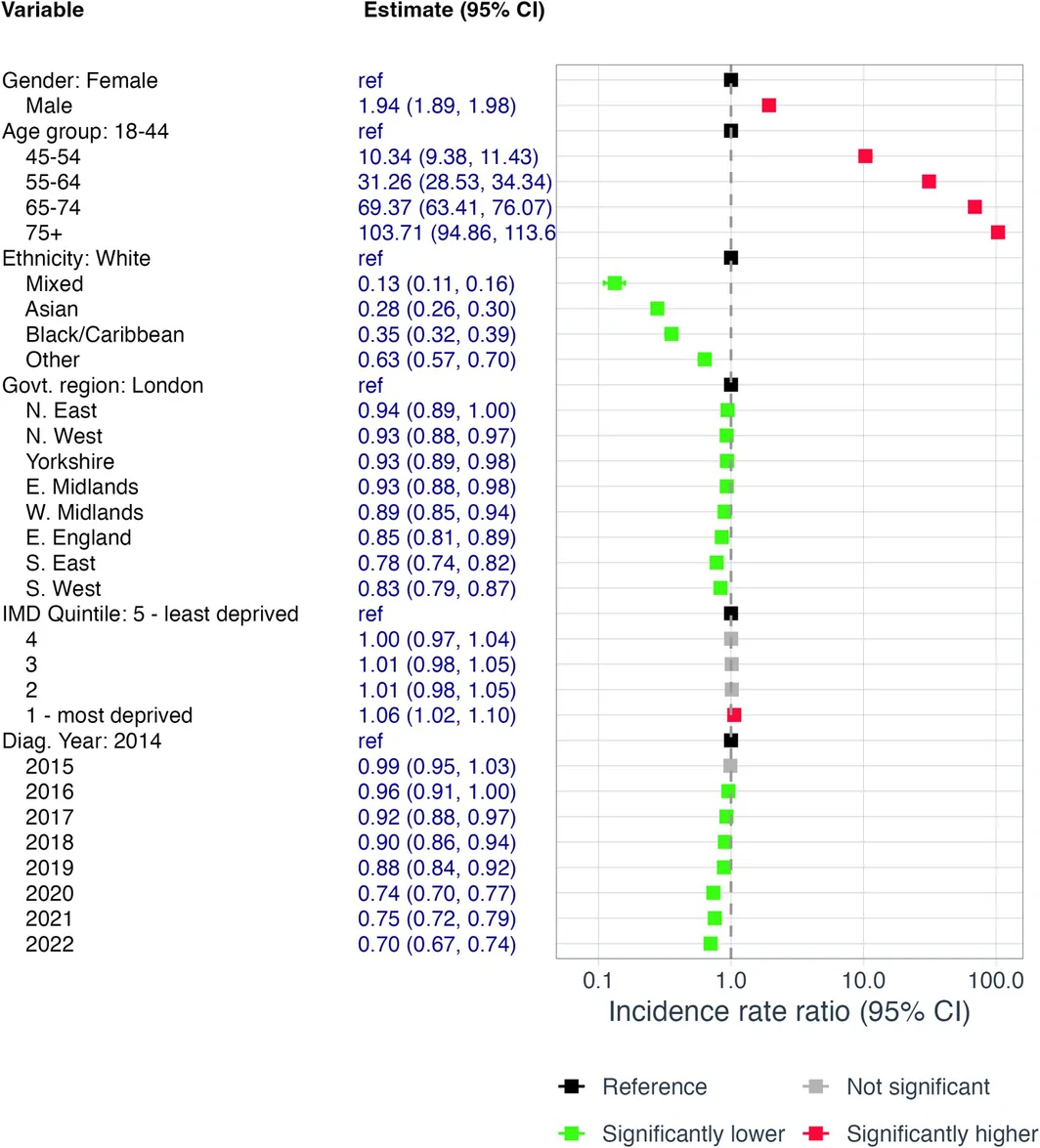
Adjusted Poisson regression models estimating the incidence rate ratios (IRRs) and 95% confidence intervals of CLL in England from 2014 to 2022. CLL, chronic lymphocytic leukaemia.

### Overall and net survival

The median follow‐up time for survival analysis was 74.4 months (95% CI 73.7–75.2), with 12 703 (36.9%) patients dying during this period. OS rates at 1, 3 and 5 years were 90.2%, 78.1% and 67.5% (Figure 3A), NS rates at 1, 3 and 5 years were substantially higher at 93.6%, 87.1% and 80.6% (Figure 3A). In keeping with this population‐based analysis, only 4685 (36.9%) deaths were attributed to blood cancer based on individual MCCDs, with a cumulative risk of dying from causes related or unrelated to blood cancer of 12.2% vs. 20.3% at 5 years (Figure 3B). Non‐haematological causes of death were predominantly infections led by COVID‐19 deaths (1076—8.4% of all reported deaths).

**FIGURE 3:**
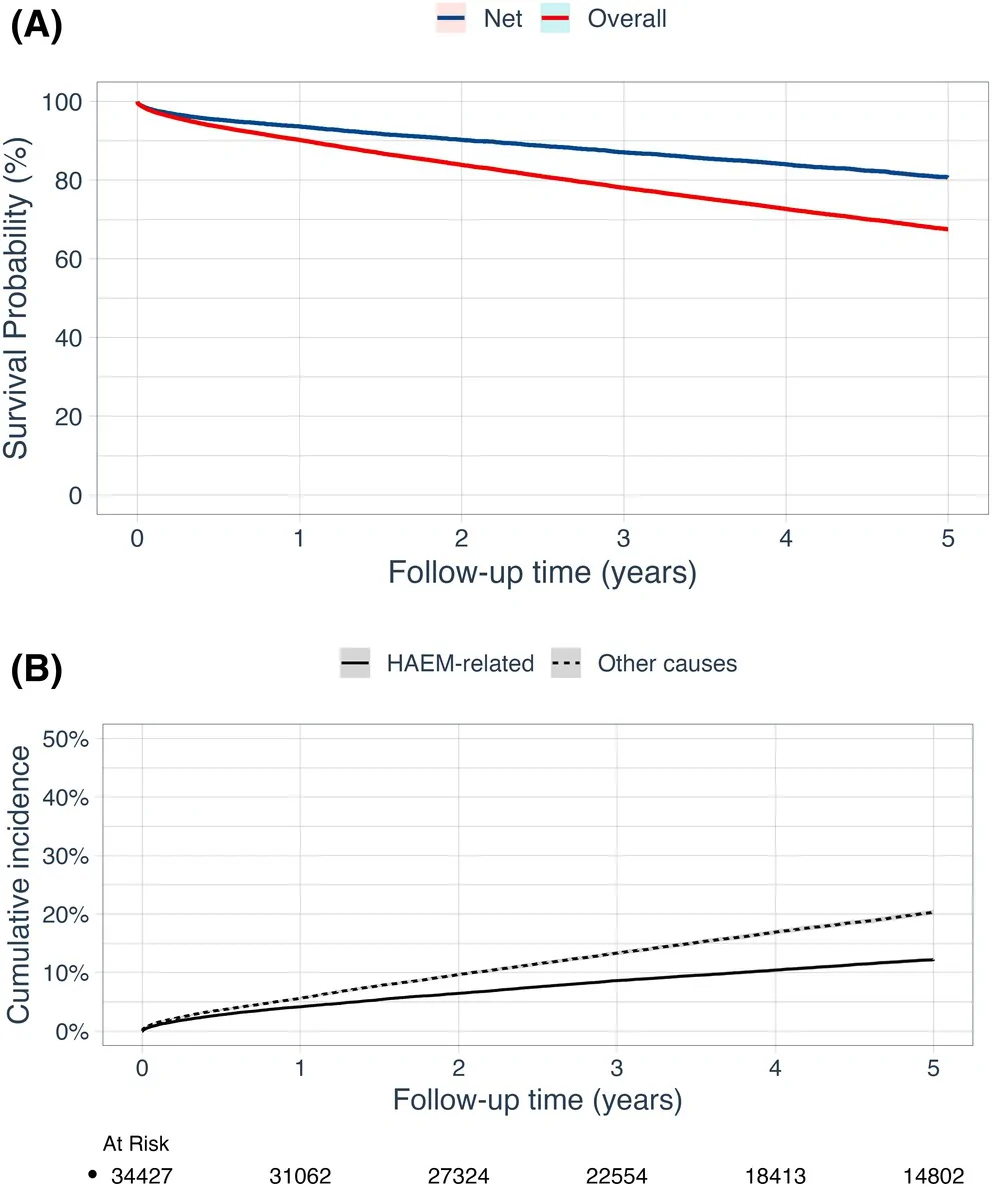
(A) Overall and net survival probabilities of CLL in England and (B) cumulative incidence of CLL mortality in England in the presence of competing risks. CLL, chronic lymphocytic leukaemia.

### Risk factors for survival

Multivariable Cox regression was performed for OS (Figure 4) and CSS (Figure 5). OS HR was higher for males versus females (1.33 [95% CI 1.29–1.38]); lower for people of Asian versus White ethnicity (0.87 [0.76–1.00]); and increased with age (15.8 [3.47–6.70] for ≥75 vs. 18–44), comorbidity (2.56 [2.40–2.74] for CCI ≥4 vs. 0) and deprivation (1.50 [1.41–1.59] for most vs. least deprived IMD quintile). A similar pattern was seen for CSS, with HR 1.17 (95% CI 1.10–1.24) for males versus females; 0.77 (0.61–0.98) for Asian versus White ethnicity; 7.94 (5.26–12.0) for age ≥75 vs. 18–44; 1.72 (1.52–1.95) for CCI ≥4 vs. 0; and 1.28 (1.16–1.41) for most versus least deprived IMD quintile. CSS also varied by government region, with HRs ranging from 0.84 (0.74–0.96) in Yorkshire and Humberside to 1.12 (0.99–1.27) in the East of England compared to London. A sensitivity analysis was performed for CSS using deaths with CLL as the cause of death, obtaining similar results to the base analysis of CSS using deaths from any blood cancer (Table S3).

**FIGURE 4:**
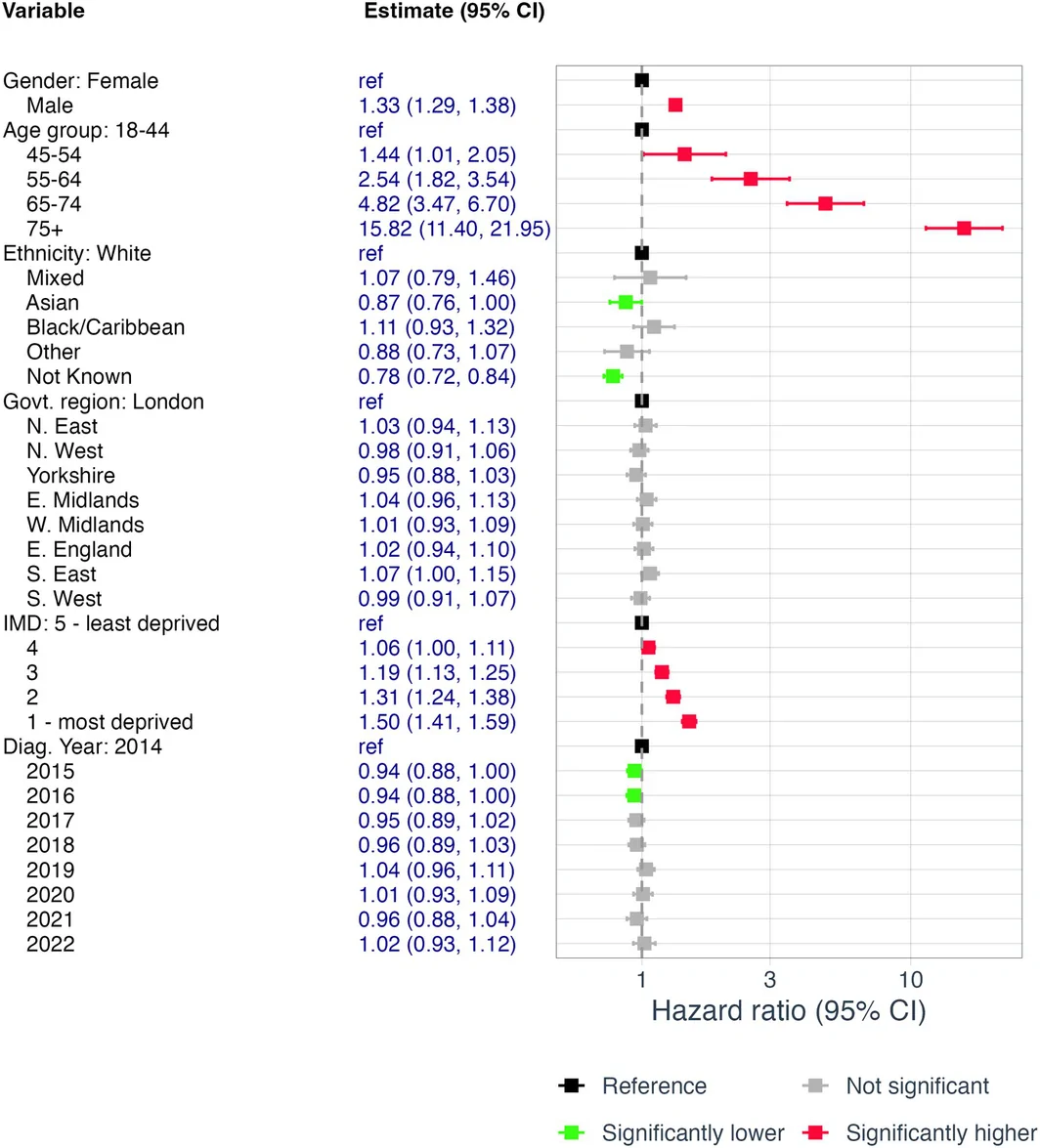
Forest plot of the multivariable Cox regression model for overall survival, showing factors associated with mortality among individuals with CLL in England. CLL, chronic lymphocytic leukaemia.

**FIGURE 5:**
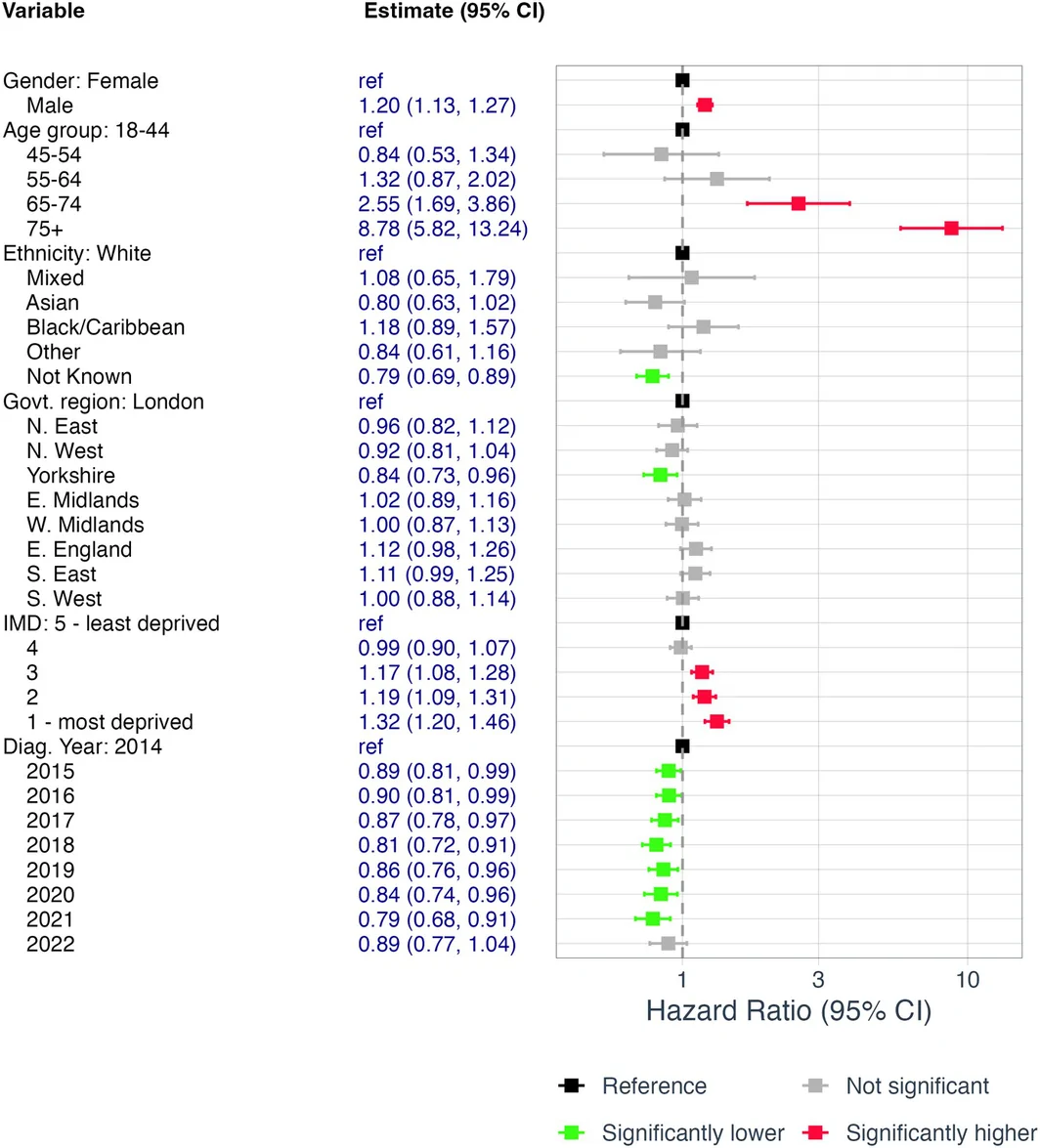
Forest plot of the multivariable cause‐specific Cox model for CLL‐specific survival in England. CLL, chronic lymphocytic leukaemia.

## DISCUSSION

We provide a timely re‐evaluation of CLL epidemiology in the context of the profound changes in CLL therapy that have taken place over the last decade. Some of the study findings were as expected, providing validation of the database and analytical methods used. Other findings were previously unreported, providing important new insights into changing patterns of CLL incidence and disparities in CLL outcomes.

The overall incidence of CLL in the present study was higher than expected from previous reports from Cancer Research UK^5^ and international data from the US (5.1/100 000),^25^ Canada (4.0/100 000),^26^ the Netherlands (3.8/100 000)^27^ and Norway (3.1/100 000).^28^ Potential explanations for this discrepancy as well as that observed for government region include differential access to health screening and differences in how cases are recorded at the boundary between early‐stage CLL and high‐count monoclonal B‐cell lymphocytosis (MBL).^29^

We observed an overall trend towards reduction of CLL incidence over time as has been shown by others.^30^ The independent association of IRR with calendar year is striking, with a 30% reduction in IRR between 2014 and 2022. The sudden drop in IRR in 2020 coincided with the first year of the COVID‐19 pandemic and likely resulted from a downturn in routine health checks and reluctance to seek medical advice for chronic symptoms. However, the IRRs were falling year on year before the pandemic and had not recovered to pre‐pandemic levels by 2022, when national restrictions were already lifted. It remains to be demonstrated if the calendar year effect reflects true reduction of the risk of CLL or else represents changing trends in health screening or diagnostic thresholds.

The association between incidence and government region was intriguing, with IRRs significantly lower for all provincial regions compared with London. Potential explanations for this observation include the possibility that people living in London have better access to health screening, resulting in a higher probability of CLL being detected at an early stage.

The identification of increasing age, male gender and White ethnicity as factors associated with CLL incidence in multivariable analysis was in keeping with previously reported population cohorts.^7^, ^25^, ^31^, ^32^ In contrast, the identification of socioeconomic deprivation as additional independent risk factor for CLL incidence is novel and unexpected, with an IRR 7% higher in most versus least deprived quintiles. A plausible explanation is that greater deprivation is associated with lifestyles, diet or exposure to antigens/mutagens that predispose the development of CLL. This hypothesis warrants further exploration.

Our study provides some interesting observations concerning the route to CLL diagnosis. Nearly one quarter of cases were diagnosed following a TWW referral, an urgent, criteria‐driven cancer referral pathway from primary care.^33^ The appropriateness of using this pathway for an indolent condition such as CLL in the context of limited healthcare resources and competing priorities may be called into question. TWW referrals may benefit from a local or national audit aimed at optimising the CLL diagnostic pathway.

Overall, CLL outcomes were encouraging, with fewer than one‐fifth of patients dying from CLL‐related causes within 5‐years of diagnosis and consistent with other reports such as the Haematological Malignancy research network (MHRN), reporting 5‐year NS of 84%.^34^ Despite our inclusive definition of CLL‐related death, the proportion of such deaths (36.9%) was lower than the rate of 40% reported in a nationwide Swedish study covering 1982–2013^8^ and 41%–44% in a nationwide Dutch study covering 1996–2020.^11^ These comparisons support the hypothesis that newer targeted treatments may be prolonging the natural history of CLL.^2^, ^35^, ^36^, ^37^ The rate of infection‐related deaths is notable, particularly those related to COVID‐19, accounting for a significant proportion of the mortality observed in this cohort, as expected during this period of observation.^4^, ^38^

Identification of increasing age, male gender and co‐morbidity as independent risk factors for shorter OS and CSS in multivariable analysis was as expected. Likewise, the identification of socioeconomic deprivation as an independent risk factor for shorter OS was unsurprising given the established association between deprivation and shorter life expectancy.^24^ However, the magnitude of the latter effect was our most significant finding, with an adjusted risk of death from any cause being 42% higher in the most deprived IMD quintile compared to the least deprived, and a progressive increase in the risk of mortality throughout the IMD quintiles. Crucially, this gap was not simply due to higher background mortality, as there was also a 28% difference in adjusted risk of death due to blood cancer.

Although social inequalities have previously been identified as a determinant of CLL outcomes in a US cohort,^39^ this is the first such report in a European population, a finding that could be explained in part by barriers to access or engagement with health services.^40^ Inequalities in CLL outcomes were further highlighted by the independent correlation of government region with CSS, raising questions of the uniformity of CLL management and care at a geographical level, independently of local deprivation factors.

Our study has strengths and limitations. Strengths include the completeness and unselected nature of the CLL dataset resulting from the mandatory cancer registration process in England; the accuracy of data borne out of the meticulous data curation carried out by NDRS, and the relevance of the study to current CLL therapy due to its recent time frame. Limitations include the paucity of staging information, which was available in only about one‐third of cases. This level of incompleteness was considered prohibitive for its inclusion in the analysis. It also highlights the need for a more assiduous approach to recording disease‐specific information, which would facilitate not only academic research but also the mapping of healthcare resource utilisation in a disease that lends itself to community‐based follow‐up. Another limitation is the limited Systemic Anti‐Cancer Therapy (SACT) data currently available in the UNCOVER database. Once this data gap has been addressed, future analyses will focus on changing patterns of CLL therapy.

In conclusion, the present study describes the largest cohort of consecutively diagnosed CLL patients in Europe^8^, ^11^ Survival data are encouraging and suggest modern treatments may be prolonging the natural history of CLL. In addition to confirming known associations, the study also made two novel observations. First, it identifies socioeconomic deprivation and government region as independent risk factors for being diagnosed with or dying from CLL that cannot be explained by differences in age, gender, ethnicity and comorbidities. Second, it shows a year‐on‐year decrease in the adjusted incidence of CLL that started before the COVID‐19 pandemic but was accelerated by it. Further studies are warranted to investigate potential causes for these trends and disparities identified and design health and social care policies that address them.

## AUTHOR CONTRIBUTIONS

**Amelia Fisher:** Writing – original draft; writing – review and editing; methodology. **Nurunnahar Akter:** Validation; methodology; data curation; software; writing – review and editing. **Shaun Bowden:** Conceptualization; methodology; writing – review and editing. **Catrin Tudur Smith:** Conceptualization; investigation; funding acquisition; methodology; validation; formal analysis; data curation; supervision; writing – review and editing. **Indrani Karpha:** Conceptualization; formal analysis; data curation; writing – review and editing. **Lelia Duley:** Conceptualization; methodology; writing – review and editing. **Debbie Yates:** Conceptualization; methodology; writing – review and editing. **Brogan Johnston:** Project administration; supervision; resources. **Hilary Lindsay:** Writing – review and editing; conceptualization; methodology. **Temitope Erinfolami:** Writing – original draft; methodology; writing – review and editing; formal analysis; data curation; validation; visualization; software. **Renata Walewska:** Writing – review and editing; conceptualization. **David Allsup:** Conceptualization; writing – review and editing; methodology. **Nicolas Martinez‐Calle:** Conceptualization; investigation; funding acquisition; writing – original draft; writing – review and editing; methodology; formal analysis; supervision. **Mark Bishton:** Conceptualization; methodology; writing – review and editing. **Toyyib Abdulkareem:** Software; data curation; formal analysis; writing – review and editing. **Yeong Lim:** Formal analysis; data curation; writing – review and editing. **Andrew Pettitt:** Conceptualization; funding acquisition; investigation; methodology; writing – review and editing; supervision; resources. **Stella Williams:** Writing – review and editing; conceptualization. **Nagesh Kalakonda:** Methodology; writing – review and editing.

## FUNDING INFORMATION

This work was performed as part of the UNCOVER health data research programme, which is funded by Blood Cancer UK (grant number 23010), North West Cancer Research (grant number C12024.10) and Clatterbridge Cancer Charity (1202412).

## CONFLICT OF INTEREST STATEMENT

NMC: Speaker and travel support from AbbVie, AstraZeneca, Janssen. Advisory Boards: AbbVie, AstraZeneca, BeOne, Takeda, CSL Behring. DA: Consultancy fees from Abbvie; travel support from BeoOne Medicines, Gilead, Sobi, CSL Behring; research support from Gilead, Roche; Member of the Board of Directors for the Daisy charity. AP: Research funding or support in kind from AstraZeneca, Celgene/BMS, Chugai, Gilead, Napp and Roche. MJB: Honoraria from AbbVie, Celltrion, Kite/Gilead, Lilly, Recordati, Roche, Takeda. Consulting or advisory role AbbVie, Incyte, Lilly, Roche; Research funding: Roche, Takeda. TE, AF, IK, YL, NA, TA, CTS, BJ, SB, HL, LD; NK: None to declare.

## ETHICS APPROVAL STATEMENT

This study was performed as part of the UNCOVER health data research programme (Research Ethics Committee approval #21/WA/0114).

## Supporting information


**Table S1.** ICD‐O‐3 codes for inclusion of CLL cases and relative distribution in dataset.
**Table S2.** Distribution of the route to diagnosis data by region.
**Table S3.** Sensitivity CSS analysis using deaths with explicit mention of CLL as the cause of death in comparison with base analysis of CSS using deaths from any blood cancer.
**Figure S1.** Map of the 9 UK regions defined by the Office for National Statistics, map generated in https://ons.maps.arcgis.com/apps/webappviewer/index.html?id=6f93241971aa47e890edcbbf209452aa and regions labelled accordingly.
**Figure S2.** Age‐standardised or age‐specific incidence of CLL by year of diagnosis in (A) the overall cohort and stratified by (B) sex, (C) age, (D) IMD quintile, (E) region and (F) ethnicity.
**Figure S3.** Cancer waiting times from referral to first diagnosis segregated by route to diagnosis.

## Data Availability

Data are available upon request. All data used in this study are stored in the Cancer Analysis System database, hosted by the NDRS. Requests for access to cancer registration data should be submitted through the NHS England Data Access Request Service via data.applications@nhsdigital.nhs.uk.
