# Supplementary material for: Trends and socio‐economic disparities in chronic lymphocytic leukaemia 2014–2022: An England‐wide cohort study from the UNCOVER group
Source: Br J Haematol. 2026 Aug 17;209(5):2056–65. doi: 10.1111/bjh.70765 (PMC13748362; doi:10.1111/bjh.70765)
Supplement: Supplementary file 1 [file BJH-209-2056-s001.docx]

**Supplementary Information**

**Table S1.** ICD-O-3 codes for inclusion of CLL cases and relative distribution in dataset

| **ICD-O-3 Codes** | **ICD-O-3 Description** | **n** | **%** |
| --- | --- | --- | --- |
| 96703 | Malignant lymphoma, small B lymphocytic, NOS | 1,393 | 4.05 |
| 98233 | B-cell chronic lymphocytic leukaemia/small lymphocytic lymphoma | 33,034 | 95.95 |

**Table S2.** Distribution of the route to diagnosis data by region.

| *Region* | *Diagnostic route* | | | | | | ***Total*** |
| --- | --- | --- | --- | --- | --- | --- | --- |
|  | Emergency presentation | GP referral | Inpatient elective | Other outpatient | TWW | Unknown |  |
| London | 271 7.9 % | 748 21.8 % | 22 0.6 % | 151 4.4 % | 581 16.9 % | 1665 48.4 % | 3438 100 % |
| North East | 153 7.6 % | 447 22.3 % | 13 0.6 % | 68 3.4 % | 217 10.8 % | 1104 55.1 % | 2002 100 % |
| North West | 303 6.1 % | 1279 25.9 % | 15 0.3 % | 197 4 % | 670 13.6 % | 2466 50 % | 4930 100 % |
| Yorkshire and The Humber | 305 8.1 % | 913 24.4 % | 13 0.3 % | 146 3.9 % | 480 12.8 % | 1888 50.4 % | 3745 100 % |
| East Midlands | 235 7 % | 825 24.7 % | 25 0.7 % | 148 4.4 % | 337 10.1 % | 1776 53.1 % | 3346 100 % |
| West Midlands | 190 5.3 % | 1009 28 % | 18 0.5 % | 141 3.9 % | 418 11.6 % | 1827 50.7 % | 3603 100 % |
| East of England | 280 7.1 % | 978 24.8 % | 27 0.7 % | 151 3.8 % | 540 13.7 % | 1973 50 % | 3949 100 % |
| South East | 378 7 % | 1469 27 % | 58 1.1 % | 242 4.5 % | 464 8.5 % | 2820 51.9 % | 5431 100 % |
| South West | 286 7.2 % | 1069 26.8 % | 26 0.7 % | 181 4.5 % | 328 8.2 % | 2093 52.5 % | 3983 100 % |
| ***Total*** | 2401 7 % | 8737 25.4 % | 217 0.6 % | 1425 4.1 % | 4035 11.7 % | 17612 51.2 % | 34427 100 % |

**Table S3**. Sensitivity CSS analysis using deaths with explicit mention of CLL as the cause of death in comparison with base analysis of CSS using deaths from any blood cancer.

|  |  | **CSS: All HAEM-related** |  |  | **CSS: CLL-related** |  |
| --- | --- | --- | --- | --- | --- | --- |
| **Variable** | **HR** | **95% CI** | **p-value** | **HR** | **95% CI** | **p-value** |
| **Gender** |  |  | <0.001 |  |  | <0.001 |
| Female | — | — |  | — | — |  |
| Male | 1.17 | 1.10, 1.24 |  | 1.17 | 1.10, 1.26 |  |
| **Age** |  |  | <0.001 |  |  | <0.001 |
| 18-44 | — | — |  | — | — |  |
| 45-54 | 0.83 | 0.52, 1.32 |  | 1.10 | 0.59, 2.05 |  |
| 55-64 | 1.27 | 0.83, 1.94 |  | 1.83 | 1.02, 3.26 |  |
| 65-74 | 2.40 | 1.59, 3.63 |  | 3.38 | 1.91, 5.99 |  |
| 75+ | 7.94 | 5.26, 12.0 |  | 12.5 | 7.08, 22.1 |  |
| **Ethnicity** |  |  | 0.006 |  |  | 0.028 |
| White | — | — |  | — | — |  |
| Mixed | 1.09 | 0.66, 1.82 |  | 1.33 | 0.79, 2.25 |  |
| Asian | 0.77 | 0.61, 0.98 |  | 0.69 | 0.52, 0.92 |  |
| Black/Caribbean | 1.20 | 0.91, 1.60 |  | 1.27 | 0.93, 1.75 |  |
| Other | 0.83 | 0.60, 1.15 |  | 0.90 | 0.63, 1.29 |  |
| Not Known | 0.83 | 0.73, 0.94 |  | 0.91 | 0.79, 1.05 |  |
| **Region** |  |  | <0.001 |  |  | <0.001 |
| London | — | — |  | — | — |  |
| North East | 0.95 | 0.81, 1.11 |  | 0.90 | 0.75, 1.08 |  |
| North West | 0.92 | 0.81, 1.04 |  | 0.92 | 0.80, 1.07 |  |
| Yorkshire and The Humber | 0.84 | 0.74, 0.96 |  | 0.88 | 0.75, 1.02 |  |
| East Midlands | 1.02 | 0.89, 1.16 |  | 1.06 | 0.91, 1.24 |  |
| West Midlands | 1.00 | 0.88, 1.14 |  | 1.05 | 0.91, 1.22 |  |
| East of England | 1.12 | 0.99, 1.27 |  | 1.12 | 0.97, 1.30 |  |
| South East | 1.12 | 0.99, 1.26 |  | 1.18 | 1.03, 1.35 |  |
| South West | 1.02 | 0.89, 1.15 |  | 1.00 | 0.87, 1.16 |  |
| **IMD Quintiles** |  |  | <0.001 |  |  | <0.001 |
| 5 - least deprived | — | — |  | — | — |  |
| 4 | 0.98 | 0.90, 1.06 |  | 1.00 | 0.90, 1.10 |  |
| 3 | 1.15 | 1.06, 1.25 |  | 1.18 | 1.07, 1.30 |  |
| 2 | 1.17 | 1.07, 1.28 |  | 1.19 | 1.07, 1.32 |  |
| 1 - most deprived | 1.28 | 1.16, 1.41 |  | 1.35 | 1.21, 1.51 |  |
| **Charlson Comorbidity Index** |  |  | <0.001 |  |  | <0.001 |
| 0 | — | — |  | — | — |  |
| 1 | 1.23 | 1.14, 1.33 |  | 1.26 | 1.15, 1.37 |  |
| 2 | 1.23 | 1.12, 1.36 |  | 1.22 | 1.09, 1.36 |  |
| 3 | 1.57 | 1.39, 1.77 |  | 1.55 | 1.34, 1.78 |  |
| 4+ | 1.72 | 1.52, 1.95 |  | 1.73 | 1.50, 2.00 |  |
| **Diagnosis Year** |  |  | 0.002 |  |  | 0.036 |
| 2014 | — | — |  | — | — |  |
| 2015 | 0.89 | 0.81, 0.99 |  | 0.91 | 0.81, 1.01 |  |
| 2016 | 0.89 | 0.80, 0.98 |  | 0.88 | 0.78, 0.99 |  |
| 2017 | 0.85 | 0.77, 0.95 |  | 0.87 | 0.77, 0.99 |  |
| 2018 | 0.79 | 0.71, 0.89 |  | 0.84 | 0.73, 0.95 |  |
| 2019 | 0.84 | 0.75, 0.94 |  | 0.85 | 0.74, 0.97 |  |
| 2020 | 0.82 | 0.72, 0.94 |  | 0.81 | 0.69, 0.94 |  |
| 2021 | 0.77 | 0.67, 0.89 |  | 0.76 | 0.65, 0.89 |  |
| 2022 | 0.88 | 0.75, 1.02 |  | 0.89 | 0.75, 1.05 |  |

**Figure S1.** Map of the 9 UK regions defined by the Office for National Statistics, map generated in <https://ons.maps.arcgis.com/apps/webappviewer/index.html?id=6f93241971aa47e890edcbbf209452aa> and regions labelled accordingly.

Regions

1) North East
2) North West
3) Yorkshire and the Humber
4) East Midlands
5) West Midlands
6) South West
7) South East
8) East of England
9) London

**Figure S2**. Age-standardised or age-specific incidence of CLL by year of diagnosis in (A) the overall cohort and stratified by (B) sex, (C) age, (D) IMD quintile, (E) region, and (F) ethnicity.

**Figure S3**. Cancer waiting times from referral to first diagnosis segregated by route to diagnosis.

2WW: Two-week way cancer pathway, GP: General Practitioner. Absolute numbers of each category are provided in Table S2.
